# Misperceptions of patients and health workers regarding malaria elimination in the Brazilian Amazon: a qualitative study

**DOI:** 10.1186/s12936-019-2854-3

**Published:** 2019-07-04

**Authors:** Felipe Leão Gomes Murta, Maxwell Oliveira Mendes, Vanderson Souza Sampaio, Abrahim Sena Baze Junior, Ximena Pamela Díaz-Bermúdez, Wuelton Marcelo Monteiro, Marcus Vinícius Guimarães Lacerda

**Affiliations:** 1 Fundação de Medicina Tropical Dr. Heitor Vieira Dourado (FMT-HVD), Manaus, Brazil; 2 Universidade do Estado do Amazonas (UEA), Manaus, AM Brazil; 3 Departamento de Saúde Coletiva da Faculdade de Ciências da Saúde., Universidade de Brasília (UnB), Brasília, DF Brazil; 4 Fundação Oswaldo Cruz, Instituto Leônidas e Maria Deane (FIOCRUZ-Amazonas), Manaus, Brazil

**Keywords:** Social perceptions, Health workers, Patients, Malaria elimination, Qualitative methods, Acceptability

## Abstract

**Background:**

Brazil has considerably reduced the number of cases of malaria in recent years and aims to eradicate the disease completely, however, vivax malaria continues to be a major challenge for the health system. In this context, the key to building a successful elimination programme may lie in the knowledge and the perceptions of the health agents, the patients affected by the disease and the personnel responsible for malaria diagnosis, treatment and control at the local level.

**Methods:**

A qualitative study was undertaken in Manaus, capital of the state of Amazonas, Western Brazilian Amazon, using a sample of 33 patients who were being treated for malaria and 22 health agents who were working in malaria diagnosis, treatment and control at the local level. A semi-structured interview with a further thematic analysis was performed.

**Results:**

There are still many myths regarding malaria transmission and treatment that may hinder the sensitization of the population of this region in relation to the use of current control tools and elimination strategies, such as mass drug administration (MDA). Most health professionals reported that the abandonment of treatment for malaria by the patient is related to poor social conditions, the high incidence of alcoholism and the low level of schooling of the affected population. One negative perception, observed in both groups with regard to the use of insecticide-treated nets, is that they may cause skin irritations and allergies. Most patients and health professionals have described malaria as an impossible disease to eliminate because it is intrinsically associated with forest landscapes, and according to them, elimination would only be possible if there were a vaccine against malaria.

**Conclusion:**

In the Amazon, cultural perceptions about the etiology of this disease can influence the behaviour and practices that social groups adopt in relation to the different forms of living in a malaria-endemic context. Negative perceptions about malaria elimination can act as a barrier when trying to reach proper coverage for control strategies such as mosquito nets and MDA.

**Electronic supplementary material:**

The online version of this article (10.1186/s12936-019-2854-3) contains supplementary material, which is available to authorized users.

## Background

Malaria elimination is a global effort and a commitment undertaken by various sectors of society and nations. In 2016, the World Health Organization (WHO) estimated that US $2.5 billion were spent on malaria control and elimination, with a small increase in funding since 2010 [1]. Despite this financial effort, there are still gaps in knowledge and barriers that hinder the implementation of malaria elimination programmes in many endemic countries. Brazil has progressed in the reduction in the number of cases, but this country still registers 37% of all malaria cases in South America [2]. The country has also implemented a falciparum malaria elimination programme in order to comply with its Sustainable Development Goals (SDGs) by 2030 [3]. Malaria in Brazil is concentrated in the Amazon region, which is characterized by structural poverty. This environment and structural poverty are both conducive to vector reproduction, and affect the most vulnerable population in the country in respect to access to health systems [4]. The changes in the dynamics of infection that have occurred in the last few years, with the predominance of vivax malaria, constitute a challenge in regard to the elimination of the disease in Brazil. Symptomless sub-microscopic infections are important in the maintenance of endemics, and their adherence to control programmes may be limited because the patients do not think they are ill [5, 6]. Currently, control measures are focused on the vector and treatment of patients with malaria, and long-term health policies are developed when there are imminent outbreaks, but after the problem has been solved, these measures are abandoned. In this regard, the elimination of malaria will require social actions in order to raise population awareness and community engagement so as to increase adherence to integrated strategies, in addition to financial investments in control tools [4].

The population’s perception of a health problem is the key factor for the construction and acceptance of control measures. Population mobilization has been described as a potential strategy for eliminating preventable diseases, such as African trypanosomiasis, dracunculiasis and schistosomiasis [7–10]. The imminent eradication of dracunculiasis shows that if perceptions about transmission and the long incubation period had not been altered, the current programme would not have been as successful. The perceptions about the disease were only modified after a process of empowerment of the affected populations through individual and collective health education which in turn caused behavioural change [8, 11]. In schistosomiasis, the misperceptions about places where transmission takes place leads the populations of endemic areas to expose themselves to contaminated water, thus hampering disease control [9, 12]. Studies of the affected population’s perceptions about malaria risks suggest that, for some groups, the disease has lost social concern, a kind of fatigue after long periods of battling against it with no solution. These perceptions are associated with the parasite species, especially with the occurrence of low severity caused by non-falciparum *Plasmodium* species [13, 14]. In tropical regions where different infectious diseases are prevalent, especially in populations with low socio-economic status, the concern for an illness tends to be of importance for these people. The more complicated and disabling disease the more worrisome it becomes [14]. This fact was observed in an area of low malaria prevalence in Zanzibar where healthcare providers and residents presented fear regarding the return of high malaria rates and, for this reason, used the protective measures [15].

Some authors studied residents’ perceptions about the strategies used to control the disease in malaria-endemic areas. In Ecuador, greater population adherence to the use of insecticide-treated nets was only achieved when they perceived immediate protection against mosquitoes [16]. Misuse of long-lasting insecticidal nets in Ethiopia was related to several factors, such as misperceptions about the purpose and durability of the product and sociocultural conditions [17]. A malaria testing and treatment programme in Zambia showed that people who did not join the programme thought the blood collected would be used for rituals or to be tested for HIV. Fortunately, those misconceptions can be altered with systematic plans of sensitization [16]. In populations vulnerable to poverty-related diseases, such as indigenous and other traditional populations, studies of perceptions about health are scarce, however they are essential for building policies that promote an intercultural dialogue between the official health system and the target populations in order to increase adherence to official treatment regimens [18]. Regarding drug-based strategies for disease elimination, misconceptions within mass drug administration (MDA) target populations contribute to a lack of initial compliance and ongoing adherence to the drug regimen, and thus become barriers to success [17]. Specific misconceptions attributed to those implementing MDA campaigns include: the lack of trial justification to the community, community misunderstanding of the medicine being administered, which group exactly is at risk of the disease, and the need to target asymptomatic carriers. Although there are only a few reports on MDA, campaigns should be targeted in order to increase success within the community, some suggest that community education and health worker training are essential [18].

Besides the affected population, the strong commitment of health professionals and convictions regarding the relevance of continuity of control and surveillance actions are essential in the process of disease elimination. In Brazil, microscopists and health agents are responsible for the diagnosis and treatment of most malaria cases. The health department is also responsible for vector control via the use of indoor residual spraying and long-lasting insecticide-treated bed net (LLIN) distribution [19]. Therefore, participation of staff in discussions and in the design of an elimination programme can generate empowerment and awareness for the cause. Health workers should be valued since listening to what the affected population has learnt about malaria should be part of the design of the malaria elimination programme. The concern is that, unless the programme makes sense to them, they will not be a fully active part of the solution of this persistent public health problem [20].

This study investigates patients’ and health workers’ perceptions about malaria and elimination measures in the Brazilian Amazon.

## Methods

### Study area and recruitment of participants

This study was performed in Manaus, the capital of Amazonas State, a malaria-endemic city located in the Western Brazilian Amazon. In 2017, the estimated population of Manaus was 2,130,264 inhabitants. In the same year, 13,595 malaria cases were recorded, however they occurred mostly on the outskirts of the city [20, 21]. Patients were recruited for interviews at the *Fundação de Medicina Tropical Dr. Heitor Vieira Dourado* (FMT-HVD), from June 2016 to December 2017. This institution is a state referral centre for malaria diagnosis and treatment and was responsible for the diagnosis of 3845 (28.3%) of malaria cases in Manaus in 2017. Records show that 98.5% of malaria cases were caused by *Plasmodium vivax*. Patient interviews were conducted at the hospital and information regarding gender, age, schooling, area of residence (urban or rural), occupation and infecting *Plasmodium* species was obtained from the patients.

As well as FMT-HVD, there are a total of 13 health clinics, distributed within the areas with a high incidence of malaria, and are responsible for diagnosis and treatment. Seven of these health clinics were visited by the research team for qualitative evaluations throughout the year of 2017. The information collected from health workers was in relation to gender, age, schooling, quantity of work experience, specific function at the malaria clinic. Health workers’ interviews were conducted at their workplace. Data collection was stopped when no new information was available [21].

### Study design

The study was a qualitative cross-sectional study, and one of its aims was to condense single statements of experience into overarching concepts by textual analysis of the obtained transcripts. By comparing comprehension of each researcher, a common agreed understanding may be achieved [22]. A semi-structured interview guide was developed comprising 22 open-ended questions and additional follow-up questions that allowed the interviewer to probe more deeply the perceptions of participants about malaria and its elimination [23]. The questions were developed after discussions within the interdisciplinary research team, which has had experience with children and adolescent patients.

Via purposive sampling, 33 patients diagnosed with malaria via thick blood smears were included in the study. These patients were interviewed on their second visit, about 7 days after diagnosis. Also included in this study were 22 health workers who work at the malaria clinics. One of the investigators, with prior experience in qualitative research, conducted in-depth interviews (IDIs). The interviews lasted approximately 40 min and took place in a quiet and comfortable room, were recorded for later transcription, and each was carried out until it was considered they had reached theoretical saturation. The guide developed for IDIs was previously tested and validated in a smaller sample by the researchers. The results and methods report follows a consolidated criteria for reporting qualitative research [24].

### Data analysis

The recording of the interview was transcribed and inserted into the MAXQDA10 program. Qualitative analysis was done through thematic framework analysis, and categories were created after previous reading of the interview transcripts. These categories emerged during the analysis process and were discussed among the researchers for consensus. Additionally, two researchers developed a codebook and started line-by-line coding.

## Results

### Social characteristics of participants

#### Patients

In cooperation with the FMT-HVD, 33 patients were recruited, who had been diagnosed with malaria and were at the final stage of treatment. The youngest patient was 10 years old and the oldest patient was 61 years old. Only one patient had been diagnosed with *Plasmodium falciparum*, while the others had been diagnosed with *P. vivax*, which is the most common species in the region. The study also indicated that the majority of patients (90.9%) reside in urban areas (Table 1).

**Table 1:** Characteristics of the 33 malaria patients and 22 health workers interviewed

Characteristics	Patients	Health workers
Number	33	22
Gender		
Male	18	12
Female	15	10
Age group in years		
10–19	2	0
20–29	6	0
30–39	9	9
40–49	10	12
50–59	5	1
> 60	1	0
Schooling in years		
1–4	3	0
5–8	10	4
9–12	15	15
> 12	5	3
Area of residence		
Urban	30	18
Rural	3	4
Plasmodium species		
*P. vivax*	32	N/A
*P. falciparum*	1	N/A
Professional status		
Employed	21	22
Unemployed	12	0
Malaria experience in years		
1–5	N/A	1
5–9	N/A	1
10–14	N/A	7
> 15	N/A	13
Job function		
Administrative function	N/A	7
Microscopist	N/A	4
Field agent	N/A	8
Driver	N/A	3

#### Community health workers

Interviews were performed with 22 health workers who agreed to participate in the research. The period of professional experience in the control of malaria ranged from one to 18 years and the majority of the interviewees (90.8%) had more than 10 years of experience (Table 1). Some of these workers have a permanent contract of employment while others are hired through political appointments, and this latter fact justifies a lack of job stability. The main activity carried out by these professionals is the passive detection of malaria cases by thick smear, followed by vector control.

**Fig. 1:**
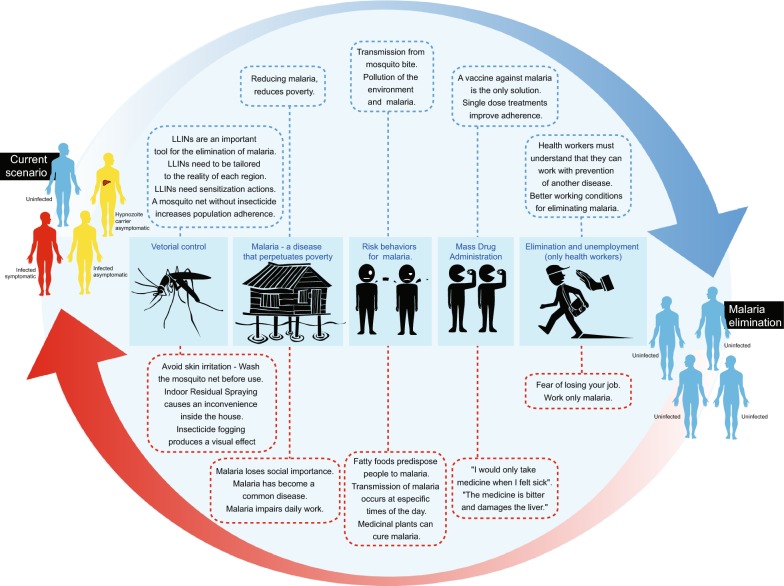
Major findings of the study when moving from control to elimination programmes

**Fig. 2:**
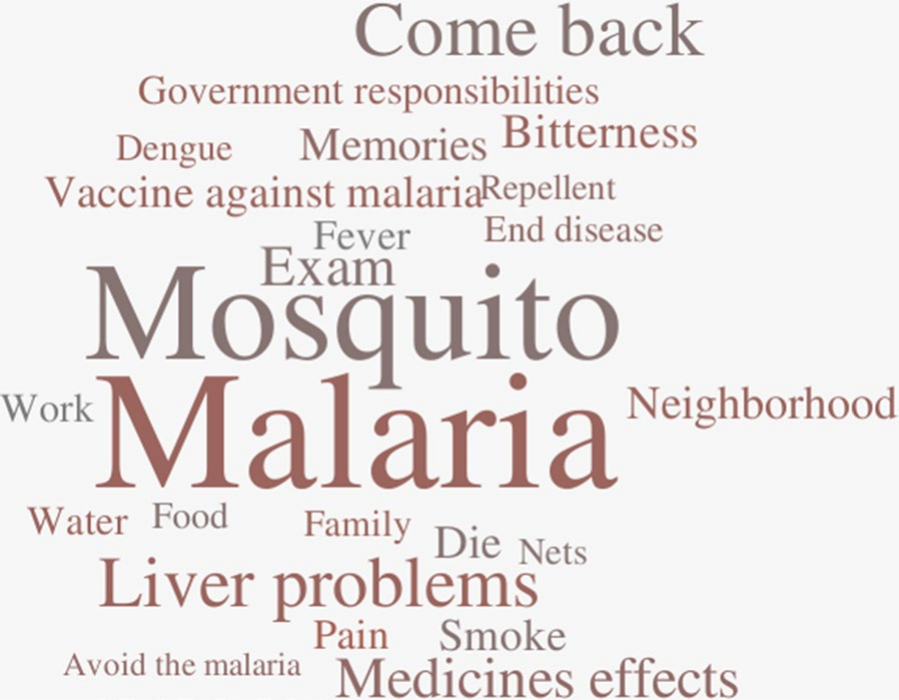
Word cloud containing 24 words or expressions most used by study participants

The analysis of the content of the patients’ and health workers’ interviews led to the identification of six main topics and the evocation rates and most frequent words and terms were computed, which are described in Figs. 1 and 2. In addition to this, a video documentary was produced and shows the reality of vivax malaria in everyday life of the riverine population and health workers in the Amazon (see Additional file 1).

### Theme 01—Risk behaviour for malaria

Some health workers, as well as some patients with malaria, believe that the consumption of fatty foods predisposes a person to malaria. In this regard, health workers, despite knowing that the transmission of the disease is caused by mosquito bites, associated worsening of the clinical condition of patients with malaria to the consumption of this type of food. For patients and some health professionals, there is also a belief that transmission of the disease can occur through the ingestion of contaminated water.

*“People say they cannot go out in the sun and mist and also cannot eat greasy food, I think that’s why I caught malaria. I ate greasy food these days and then I felt a kick, I felt that warmth in the body.”* (Patient, participant 02)

*“I think I got malaria because I went down to the stream and drank the water there. I was there taking a shower and accidentally drank this water.”* (Health worker, participant 27)

Although all health professionals and most patients recognize that transmission occurs from a mosquito bite, there is a perception that disease can be transmitted through interpersonal contact or body fluids

*“Malaria is picked up through the mosquito bite, but I do not know exactly the name of the mosquito. I also think that you get it through contact with a contaminated person who has malaria, maybe touching contaminated blood of that person we can get the virus too.”* (Patient, participant 23)

All the patients and health professionals mentioned that the transmission of malaria occurs at specific times: at dawn (05.00 to 06.00) and at dusk (17.00 to 20.00).

Health staff reported that the main problem in adhering to malaria treatment comes from patients who are chemically dependent, especially alcoholics. According to them, this creates a problem in malaria control and elimination strategies because these patients keep the disease in certain regions. They complain of prolonged treatment and adverse effects of the drug and discontinue treatment after a few days, when there is improvement in symptoms.

*“Here, we have many cases of treatment withdrawal due to alcohol intake, the patient takes the first doses and gives up treatment to drink alcoholic drinks.”* (Health worker, participant 08)

*“Nearby, there is a community called the Colosseum where many alcoholics and drug addicts live. There they contract malaria and end up abandoning the treatment on the third dose because they already feel well, and this generates new cases of malaria because they are not fully cured.”* (Health worker, participant 03)

The majority of patients and health professionals believe in alternative treatment via the consumption of medicinal plants, such as the use of lemon grass tea (*Cymbopogon citratus*) and tea made from carapanaúba (*Aspidosperma nitidum*) tree bark for curing malaria.

### Theme 02—Malaria—a disease that perpetuates poverty

The survey respondents believe that malaria is closely related to poverty and social inequalities. One of the problems reported is the loss of jobs as a result of the disease, especially in employment in which workers need to use their physical strength.

*“What makes me sad is seeing the residents suffer from the disease. I have seen very poor people suffering from malaria and I think it is an illness that has an efficient treatment and the government does not pay much attention to this type of situation, especially for the rural resident.”* (Health worker, participant 08)

*“Malaria carries many disadvantages for patients because they cannot work and as most of them have no schooling, then their financial situation gets much more complicated when they are sick.”* (Health worker, participant 05)

*“I lost my job because of malaria. At that time I worked without a work contract in a recycling factory and because the work was heavy and I had malaria, the boss sent me away.”* (Patient, participant 25)

For health workers, another major problem in the control of malaria is the precariousness of food and health conditions associated with poverty.

*“I remember the story of a boy who must have been about two years old and had malaria. I went to make the visit and when I arrived there were the three brothers who were sitting down and all three were eating clay because they had nothing to eat. I’ll never forget this. I kept thinking how could I give the medicine which is strong without the patient being fed?”* (Health worker, participant 15)

According to the professionals, malaria is an important problem for patients, but when compared to the daily problems associated with poverty and other diseases such as dengue and leishmaniasis, it loses social importance. Today, for most participants (heath workers and patients), malaria is as common a disease as flu, and epidemiological data indicates that in recent years Brazil has reduced malaria incidence and mortality.

However, stories of death caused by malaria were highlighted in the reports from all the interviewees. Patients and health workers reported memories of a time when they feared malaria because it killed a lot of people.

*“My brother died of malaria. He was 7* *years old. He started with pain and malaise. When he got worse, there was no saving him, the pills he took did not take effect anymore because his malaria was already very strong. My mother was devastated.”* (Patient, participant 05)

*“I have already lost seven brothers, so my mother tells me that she was a little afraid for us because of what happened to our other brothers could happen to us, too. She never really explained to us what happened, she just said they had a fever, cold, and it was malaria. Soon after, they transferred to the nearest hospital that was in Humaitá, but it was a day*-*long trip and half the people died on the way because they did not get to the hospital in time*.” (Patient, participant 11)

### Theme 03—Vector control: bed nets or bad nets, indoor residual spraying and space spraying

The use of LLINs is an important strategy that has been adopted by the government for the elimination of malaria in Brazil. In the state of Amazonas, in 2016, 40,000 LLINs were distributed. However, most of the patients interviewed did not realize the importance of LLINs for protection against malaria. Most patients believe that malaria is transmitted outside the house and at specific times, so for them LLINs would not be effective. In addition, many have reported the skin irritation and allergies caused by the insecticides used in the nets as an important factor for not using the nets. Some patients even washed their mosquito nets several times before using them.

*“We protected ourselves with mosquito nets, but they gave us an allergic reaction because my sister*-*in*-*law gave us her new ones, only she did not explain how they should be used, she didn´t explain to me that you had to wash them first, because as they come with poison and it causes an allergy”.* (Patient 15)

*“Where we used to live, we used LLINs a lot. It is not very good, I remember that my mother put the net up and I was short of breath and, when the mom closed the room, I took it down, it was a child thing, the LLINs stifled my breath.”* (Patient 20)

According to health workers, LLINs are an important tool for the elimination of malaria, but LLINs need to be adequate to the reality and climatic conditions of the region. They point out that the biggest complaint of the population was about the occurrence of allergies caused by the insecticide. Nevertheless, they believe that an increase in the adhesion to the LLINs would require effective actions of sensitization, since there, are several reports of patients who did not want to use them and, after the health education, they began to use them. Some health workers believe that washing LLINs for insecticide removal increases population adherence.

*“Many patients do not like the net because of the insecticide, because it has a strong insecticide which is itchy and causes allergy, but I believe with health education action we created the acceptance.”* (Health worker, participant 10)

*“These mosquito nets that were delivered came with insecticide and people got allergies, and they complained about that. We recommend that they wash the mosquito nets with soap so that the insecticide is removed and they could use them.”* (Health worker, participant 11)

In the opinion of the health workers, indoor residual spraying (IRS) represents, for most of the patients, a problem because causes an inconvenience inside the house and necessitates the temporary removal of furniture. The people interviewed perceived an improvement in the control of other insects like flies and cockroaches, but they claim that it is only slightly effective against the mosquito. According to these workers, another reported problem is the allergy caused to some residents by the insecticide, which contributes to the non-acceptance by the community. Professionals perceive that the population prefers insecticide fogging because it visually causes an impact that the spray does not have.

*“I think that the fogging (insecticide) does not only remove the malaria infected mosquito, but it keeps away other insects and so they like it.”* (Health worker, participant 01)

*“They like the “smoke”, because it is a visible thing and when they see that smoke they like it, because, in an area that has a lot of mosquitoes, the smoke seems to remove them. So it’s an immediate thing and they think it’s good and they even ask us to make the smoke.”* (Health worker, participant 16)

### Theme 04—Mass drug administration

When asked to comment on a hypothetical MDA programme, 18 patients were supportive of the idea while 15 patients opposed. The main argument against MDA was fear of problems because of the use of medicines. The need to feel sick to take the medication was one of the arguments presented as opposition to the MDA. However, when asked about their attitude in a hypothetical situation, where the malaria result was positive, but they were asymptomatic, the majority of these patients were in favour of taking medication. The patients’ main argument against MDA was the bad experience during treatment with primaquine, mainly due to adverse reactions.

*“I think there are people at risk with this program because it depends on the person’s body because the medicine fights malaria but if the person does not have malaria that person’s liver is destroyed.”* (Patient 02)

*“No, certainly this programme would not work, because if I do not have this disease and I take medicine, it will affect some organ of mine, usually the liver and then I will have problems in my liver.”* (Patient 01)

Most health professionals do not believe in the success of an MDA campaign. According to them, the adherence of people who are not sick would be low because it does not make sense to take a medication without being symptomatic.

*“I think they would not accept taking a medicine without feeling anything, they already complain about us pricking their finger when they are not feeling sick, imagine taking this medicine.”* (Heath worker 02)

Another problem according to health professionals is the large number of pills taken by patients with vivax malaria, which is the main culprit for treatment abandonment. Therefore, for most of them, an MDA programme would not work unless the number of drugs was reduced. The lack of knowledge about glucose-6-phosphate dehydrogenase deficiency (G6PDd) was evident in all the interviews with health professionals; most of them were unaware of primaquine-induced haemolysis in G6PDd patients.

### Theme 05—Elimination

Most health workers (17/22) do not believe in the elimination of malaria in Brazil using the current tools. They affirm that the geographic conditions of the Amazon, associated with the lack of infrastructure for the work, prevent the correct control of the disease. They believe that developing a vaccine would be the ideal solution for elimination. The political effort for elimination has also been cited as central to this process, but in the perception of health workers, the government is not concerned about a disease related to poverty, except at the time of elections. Many believe it is impossible to eliminate malaria because the vector mosquito is widespread in the forest. For them, unless the forest is cleared, malaria will continue to be present in the region.

*“To end malaria, scientists would have to take it more seriously, and then create a definitive vaccine for malaria only so it would end the disease.”* (Health worker, participant 15)

*“I believe that it would not be possible to eliminate malaria in Brazil, for that, first we would have to cut down the whole forest, and that is impossible.”* (Health worker, participant 19)

*“I think it’s possible to end it but the problem is that the government does not pay much attention to this type of disease, they forget about this population that suffers from malaria and they only remember when the elections arrive.”* (Health worker, participant 09)

The perception that the disease and its vector are intrinsic to the jungle is present in the reports of most patients and for them it would be impossible to eliminate the disease in the Amazon. Similar to the perception of health workers, the discovery of a vaccine is cited by most patients as the best tool for elimination.

*“I think it’s very difficult to eliminate malaria and, from the information that I have, malaria mosquitoes are in tropical areas in the jungle which greatly facilitates the disease, so to end malaria, you would have to cut down our jungle.”* (Patient 09)

### Theme 06—Elimination of malaria and unemployment (health workers only)

When asked how their job situation would look if malaria were eliminated, five health workers were worried about losing their jobs because they did not have a permanent contract of employment. For most of them, however, the elimination of malaria would be a great victory, a result of all their daily efforts. According to them, this would be a great incentive to fight other diseases that afflict the poorest people, such as dengue and leishmaniasis.

*“If they end malaria, I would be thrown into a corner and I would not find that good. But this disease will not end! I have faith in God that malaria will never end, because I like to work with malaria, I like to prick fingers.”* (Health worker, participant 02)

*“For me, it would not be good to end malaria because I work in this area, and I would be unemployed so I do not think it’s a good thing to end malaria.”* (Health worker, participant 08)

*“In relation to my job, I would seek another solution, because I think that the people who suffer are the population and I would not want to promote myself* via *the disease of the people.”* (Health worker, participant 15)

## Discussion

Health education has, as one of its pillars, the promotion of health and sensitization of people to be responsible with their health and the health of the community where they live. In this sense, it is necessary to understand the perceptions that exist in the populations that are vulnerable to a certain disease [9, 25]. Community perceptions may differ from political perceptions and thus define popular participation in the process of eliminating an illness, as in the example of dracunculiasis [8]. In interviews, the perception risk in relation to malaria is directly associated with disease mortality, and there are many diseases afflicting this population in the Amazon so malaria, being common, has lost its social importance in the lives of the most infected people. However, the morbidity of vivax malaria is high and creates an economic burden for the poor people who are also the most infected in the Amazon. The fear of losing jobs due to malaria was shown in this study, and most health workers reported that the patients’ treatment is compromised by the precariousness of social conditions, such as insufficient nutrition and low levels of education.

Myths related to transmission are still a major challenge for health education programmes. In this study, results indicate misperceptions reported by patients and health professionals about the cause of the disease, such as transmission via the ingestion of contaminated water. In other regions, other authors report similar perceptions, which may influence risk behaviour for malaria [26–28]. Reports that fatty foods negatively affect malaria treatment were evidenced in the interviews in the two groups (patients and health workers). These reports indicate a perception of liver problems associated with the disease that would be aggravated by consumption of these foods. Similar findings have described perceptions of worse malaria symptoms associated with fat, spices and sweet foods [27]. A study in the same state showed that the teachers of the basic education network had little information about the transmission mechanisms of malaria, vectors and means of prevention and this may show how the myths about the disease persist in the population [29].

In the Amazon region, indigenous traditional knowledge has social importance and determines the behaviour of riverine communities, which maintain a strong relationship with nature. In addition to this, the results show that it is still common to search for alternative treatments, such as the use of herbal teas from the forest. This knowledge is important and should be considered in a campaign to eliminate the disease in the region because it directly affects the adherence to modern treatment. In addition, some in vitro studies show the pharmacological potential of teas or herbal remedies against forms of *Plasmodium*, which is important in the context of the spread of drug-resistant parasites [30–32].

In 2016, the Government of the state of Amazonas distributed 40,000 LLINs to populations of cities with a high malaria incidence [33]. The results indicate that negative perceptions about LLINs can influence the control of the disease when there is not good adherence by the population. In areas of high incidence of malaria, the benefit of using LLINs is evident and there are several studies showing their success as a control tactic, including in Brazil [6, 34]. However, reports of allergies and reactions to the insecticides used should be investigated and discussed in health education programmes with the population at risk, as these negative perceptions compromise the sensitization to the use. These factors must be considered before mass distribution of LLINs. The reports from the two groups interviewed show there is a preference, even a request by the population for the daily space spraying as a vector control activity. This can be explained by the visual impact of smoke and the house disturbance caused by IRS. Vector control activities need to have support of the population, however only health education can provide these people with knowledge of the environmental impact and implications for health caused by insecticides.

MDA campaigns are an important strategy for eliminating malaria and are recommended by WHO in specific situations [35]. Despite all the care taken to cover these campaigns, the adherence of people at risk is fundamental to the success of the action [36, 37]. The interviews conducted indicate a negative perception of patients and professionals in regard to this strategy. The main argument of patients is the need for a diagnostic test that proves a parasite infection. In this sense, mass screening and treatment (MSAT) and focal screening and treatment (FSAT) could be a viable strategy for the Brazilian Amazon, which could increase acceptability in the population. The study showed a lack of knowledge in the health professionals when interviewed about G6PD deficiency. In an MDA scenario, this knowledge is vital to the success of the campaign. In addition, it is urgently necessary to train these professionals in order to test patients who may have vivax malaria before primaquine administration.

Most of participants do not believe that the elimination of malaria will result from the current actions taken by the government. Patients and health professionals think that only when science develops a vaccine will malaria elimination be feasible. Studies of perceptions about malaria vaccines have shown that there is a high acceptance for this preventive tool and the population prefers this alternative to treatment via medicines [38, 39]. The not expect the population to support an elimination programme, if health workers do not believe in this programme, and the partnership between managers and health workers must include a dialogue beyond just work instructions. The planning of a malaria elimination programme in Brazil should observe the employment situation of many of these health workers, because it may be a limiting factor for the adherence of the workers and consequently of the population.

Eliminating malaria in the Amazonian context represents a major human and logistical challenge. Besides the great distances between communities that suffer from the disease, and the lack of transportation infrastructure, there are still the myths and incorrect perceptions that permeate the minds of the Amazonian population, including those of the health professionals. However, measures such as the establishment of permanent strategies for health education are capable of raising awareness among the population about the elimination of the disease. Health education also produces empowerment in the population, which allows better adherence to the control measures that are used in the fight against malaria in the Amazon.

The emergence of new medicines for the treatment of malaria, such as tafenoquine, demand a greater level of information about malaria, since the knowledge of health professionals and the affected population about G6PD deficiency is fundamental to the success and adherence to this new treatment.

A successful strategy used elsewhere and in Brazil to control neglected diseases, such as schistosomiasis and geohelminth infections, is to use schools and children as a multiplier of scientific and correct knowledge about these diseases. The government could use this same approach for malaria in the most affected regions, bringing correct information about the disease to the population via schools.

### Limitations

In this study, was adopted a qualitative approach with a purposive sampling. The study has a limitation in that it does not represent the perceptions of all patients and health workers in the Brazilian Amazon. Despite efforts to minimize interference, interviews may contain some generalizations from participants, especially when they report behaviour of other people in the community [40].

## Conclusions

There will be no elimination of malaria without social mobilization of different social actors. This means strong community engagement of the most affected social groups and political commitment at state-level for designing an opportune and applicable elimination strategy. Social determinants of health are strong markers for vulnerability to malaria and to the type of response both at individual and community level. Cultural notions about the etiology of this disease has influenced the behaviour and practices that social groups adopt in relation to the different forms of living in an endemic context for malaria. Perceptions of health professionals and patients about malaria are important for the planning of a malaria elimination programme. Through these perceptions it is possible to formulate efficient educational materials, which allow an interaction between popular and scientific knowledge.

## Additional file

**Additional file 1.** Documentary film about vivax malaria in Brazil Amazon.

## Data Availability

The data that support the findings of this study are available from Fundação de Medicina Tropical Dr. Heitor Vieira Dourado, but restrictions apply to the availability of these data, which were used under license for the current study, and so are not publicly available. Data are however available from the authors upon reasonable request and with permission of Fundação de Medicina Tropical Dr Heitor Vieira Dourado.

## Abbreviations

MDA: mass drug administration
WHO: World Health Organization
SDGs: Sustainable Development Goals
LLINs: long-lasting insecticide-treated bed net
IDIs: in-depth interview
IRS: indoor residual spraying
G6PDd: glucose-6-phosphate dehydrogenase deficiency
MSAT: mass screening and treatment
FSAT: focal screening and treatment
